# Comparisons of volumetric modulated arc therapy (VMAT) quality assurance (QA) systems: sensitivity analysis to machine errors

**DOI:** 10.1186/s13014-016-0725-4

**Published:** 2016-11-07

**Authors:** Bin Liang, Bo Liu, Fugen Zhou, Fang-fang Yin, Qiuwen Wu

**Affiliations:** 1 Image Processing Center, Beihang University, Beijing, 100191 People’s Republic of China; 2 Department of Radiation Oncology, Duke University Medical Center, PO Box 3295, Durham, NC 27710 USA

**Keywords:** Quality assurance, Volumetric modulated arc therapy, Receiver operator characteristic, Gamma analysis

## Abstract

**Background:**

In volumetric modulated arc therapy (VMAT), gantry angles, dose rate and the MLC positions vary with the radiation delivery. The quality assurance (QA) system should be able to catch the planning and machine errors. The aim of this study was to investigate the sensitivity of three VMAT QA systems to machine errors.

**Methods:**

Several types of potential linac machine errors unique to VMAT delivery were simulated in sinusoidal function of gantry angle, including gantry angle itself, MLC position and linac output. Two commercial QA systems, ArcCheck and Delta^4^, and an in-house developed EPID technique were compared in this study. Fifteen full arcs from head and neck plans were selected and modified to include five magnitudes of each type of error, resulting in measurements and γ analyses of 240 arcs on each system. Both qualitative and quantitative comparisons were performed using receiver operating characteristic (ROC), γ pass rate gradient, and overlap histogram methods.

**Results:**

In ROC analysis, the area under curve (AUC) represents the sensitivity and increases with the error magnitude. Using the criteria of 2 %/2 mm/2° (angle to agreement, ATA, only for EPID) and keeping AUC > 0.95, the minimum error detectable of ArcCheck, Delta^4^ and EPID are (2, 3, 3)° in gantry angle and (4, 2, 3) mm in MLC positions for the head and neck plans. No system is sensitive to the simulated output error, the AUC values were all below 0.70 even with 5 % output error. The γ gradient for gantry angle, MLC position and output errors are (−5.1, −2.6, −3.6)%/°, (−2.6, −7.1, −3.3)%/mm and (−0.2, −0.2, −0.3)%/% for ArcCheck, Delta^4^ and EPID, respectively. Therefore, these two analyses are consistent and support the same conclusion. The ATA parameter in EPID technique can be adjusted to tune its sensitivity.

**Conclusions:**

We found that ArcCheck is more sensitive to gantry angle error and Delta^4^ is more sensitive to MLC position error. All three systems are not sensitive to the simulated output error. With additional analysis parameter, the EPID technique can be tuned to have optimal sensitivity and is able to perform QA for full field size with highest resolution. In addition, ROC analysis avoids the choice of γ pass rate threshold and is more robust compared with other analysis methods.

## Background

Volumetric modulated arc therapy (VMAT) requires the precise synchronization of linac gantry rotation with MLC motion and radiation delivery [1]. The most significant feature of VMAT that distinguishes from intensity modulated radiation therapy (IMRT) is the gantry rotation modulation during delivery. The mechanical gantry rotation, which naturally has various gravitational effects on linac components, may trigger extra machine errors that are unique to VMAT delivery. The potential machine errors related to gantry rotation should be the focus of the investigation on VMAT quality assurance (QA).

Commercial systems such as ArcCheck (Sun Nuclear, Melbourne, FL) [2–6] and Delta^4^ systems (ScandiDos AB, Uppsala, Sweden) [3, 6, 7], are currently widely used for VMAT QA. When evaluating different VMAT QA, a natural question to be raised is: which one is more sensitive to the rotation related error during VMAT delivery? To be more specific, what is the minimum detectable error by each system?

Numerous publications have covered this topic previously [8–12]. Hauri et al. investigated the γ pass rate change after introducing errors on gantry angle during VMAT delivery using Delta^4^ [8], where the intentional error was of sinusoidal form to simulate the gravitational effect. They evaluated the change of gamma pass rate with 2° and 10° errors. For the sensitivity to MLC errors, Heilemann et al. [9] investigated Delta^4^ system and Coleman et al. [11] on ArcCheck system, respectively. The simulated errors included open, closed and shifted MLC bank errors, resulting in an enlarged, narrowed and shifted field during delivery. The error was of constant magnitude during the entire delivery. To determine the sensitivity, an extra pass rate threshold value was required in the analysis. Other machine errors on collimator rotation [6], setup error [13] and planning error [14] were also investigated by different investigators. However, the studied errors were of limited magnitude thus inadequate to fully quantify the sensitivity.

These studies provided insightful perspectives on this topic. However, a complete understanding cannot be obtained from any single publication, and the questions raised earlier are not answered thoroughly. In addition, most simulated errors are of constant magnitude which remains the same during gantry rotation. The machine errors that vary with gantry rotation should deserve more attention for VMAT QA due to the gravitation effect. Another concern is the need for extra pass rate threshold during the previous sensitivity analyses, which can be subjective to institutional bias. This can undermine the credibility of these studies.

In this study, we investigate the machine error sensitivity of three QA systems: ArcCheck, Delta^4^ and an in-house developed electronic portal image device (EPID) based technique [15]. Three types of errors unique to the VMAT delivery (gantry angles, MLC positions, and linac output) with various magnitudes were investigated. These errors are all of dynamic nature, i.e., they vary with the gantry angle. Furthermore, the results were analyzed with several independent and complementary techniques.

## Methods

In this section, we first briefly describe the QA systems studied, then present the methods to simulate machine errors and to perform sensitivity analysis.

### QA systems

**Fig. 1:**
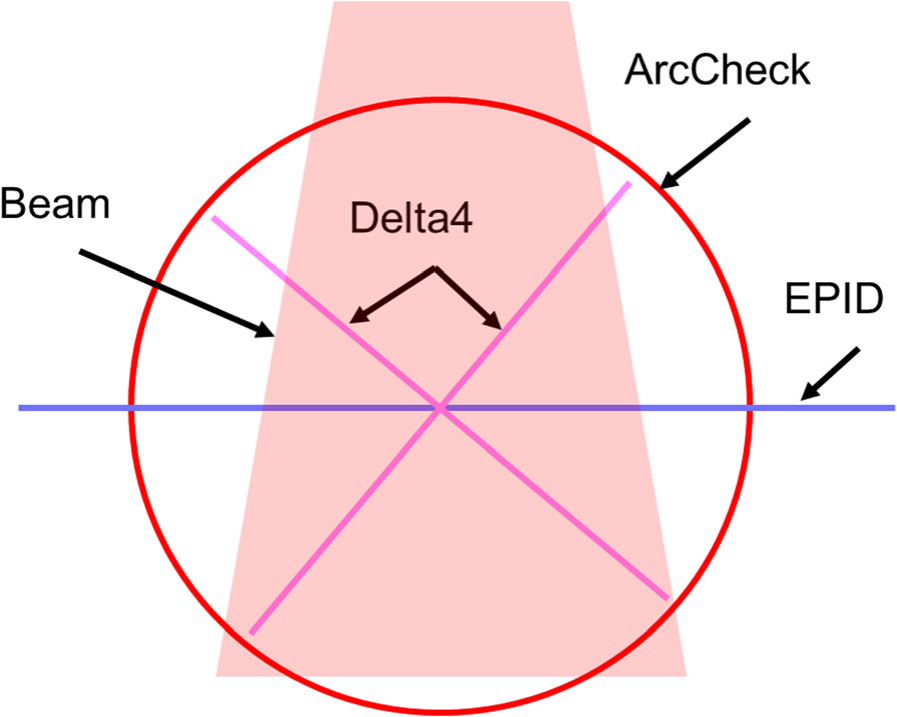
Comparison of detector layout of three QA systems

Two commercial systems (ArcCheck and Delta^4^) and an in-house built EPID based system were included in this study. Figure 1 compares the detectors layouts in these systems.

#### ArcCheck system

The ArcCheck system contains 1386 n-Si diodes placed on a cylindrical surface of 21 cm diameter inside a ring-shaped PMMA phantom. The detectors, 1 cm apart, form 21 helical continuous rings with 66 detectors on each ring. The 3D dose matrix computed by the planning system is imported and unfolded in SNC software into a 2D dose plane covering all detectors. The system acquires data at every 50 ms during treatment delivery, which are converted and accumulated to composite dose for subsequent analysis.

#### Delta^4^ system

The Delta^4^ system consists of 1069 p-type diodes on two near orthogonal planes embedded in a cylinder PMMA phantom, 40 cm in length and 22 cm in diameter. The detectors have 0.5 cm spacing in the central 6 × 6 cm^2^ region and 1 cm in the outer region. Measurements were synchronized to beam pulse and a 3D dose distribution was obtained through interpolating the measured data on the two planes. The QA analysis is performed using the calculated and the composite measured 3D dose distribution on the phantom.

#### EPID technique

**Fig. 2:**
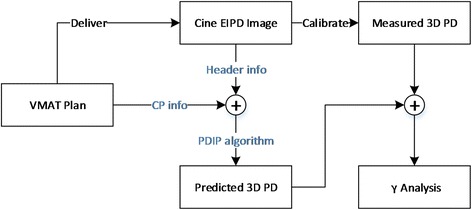
Flowchart of EPID technique

This technique utilizes EPID in cine mode [15], and the flowchart is shown in Fig. 2. The VMAT plan is delivered on a TrueBeam linac (Varian Medical Systems, Palo Alto, CA) and the acquired images are converted to portal dose (PD) after calibration and profile correction. The monitor unit (MU) and gantry information in each image header, and the control point (CP) information in the DICOM plan were used to compute a predicted PD using an in-house portal dose image prediction (PDIP) algorithm. Previous study showed that the gantry angle measured by the EPID was not accurate in Clinac 21EX [16], so it could not be used for VMAT QA without the use of an external phantom. In our previous publication [15], it was demonstrated that the gantry angles recorded on TrueBeam is accurate within 0.3° due to the use of dedicated micro-controllers in the EPID system, therefore external phantom and alignment are not necessary for the EPID QA technique on Truebeam linacs. A 3D PD is obtained by stacking up all 2D frames of PD, with the 3rd axis representing gantry angle. Global 3D γ analysis is performed using the measured and predicted 3D PD matrix. In addition to the threshold, dose difference (DD) and distance-to-agreement (DTA) criterion parameters, the γ analysis of EPID technique requires an extra criterion: angle-to-agreement (ATA). For EPID γ analysis, the DTA and ATA criteria confine the search range within and between each PD plane. Therefore, the combination of DTA in 2D and ATA on the 3rd axis forms the 3D analysis space. Detailed description of ATA can be found in Reference [15].

Improvement made in this study over previous publication [15] are the following. We independently implemented a calculation engine of Varian’s PDIP algorithm, which significantly improved QA workflow efficiency. Also, the VMAT plan is now delivered directly in DICOM format in clinical mode on Truebeam version 2.0 or higher, without the need of a research mode license as in the previous work. All software programs were written in the MATLAB environment (MathWorks, Natick, MA) except that 3D γ analysis was implemented using C/C++ for calculation efficiency. The acquired portal dose image has an area of 40 × 30 cm^2^ or 40 × 40 cm^2^ depending on the panel versions and a resolution 0.39 × 0.39 mm^2^, which was down-sampled to 0.78 × 0.78 mm^2^ in QA analysis.

### Machine error simulation

In IMRT delivery, both dose rate and MLC leaf positions are modulated to deliver a desired fluence. The main feature distinguishing VMAT from IMRT is the additional modulation in gantry rotation. Therefore, gantry angle and machine characteristics as a function of gantry angle should be checked during dynamic delivery. Another consideration on error simulation is that the simulated errors at gantry angle of 0° and 180° should be zero or small enough to escape the routine linac QA. Otherwise, the standard machine QAs usually performed at these angles could detect these errors.

**Fig. 3:**
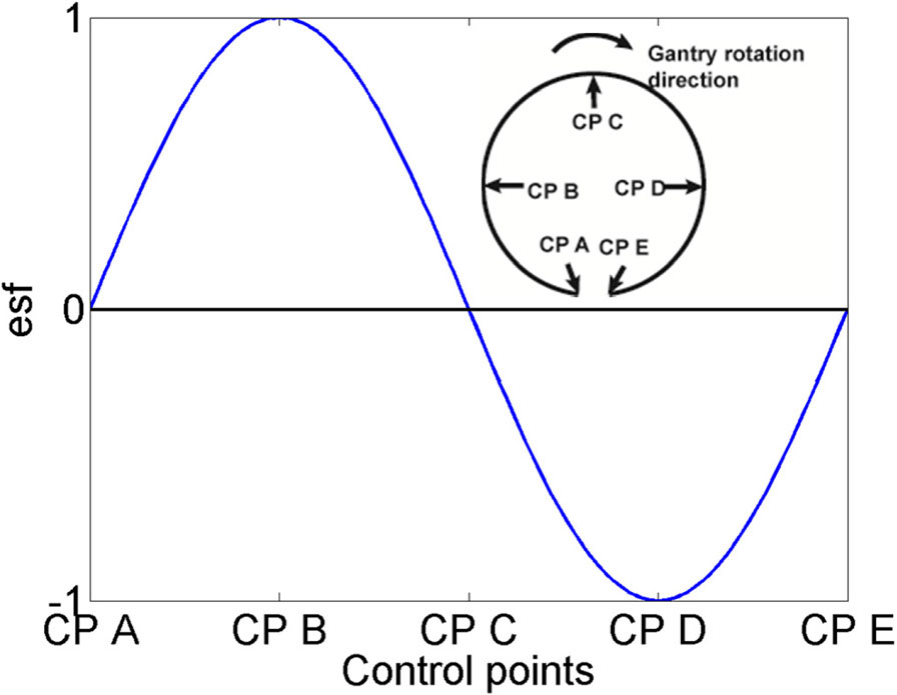
VMAT error simulation function vs. control points and gantry angles

In this study, the error simulation function (*esf*) is modeled in the sinusoidal form of the MU index of each CP (*MU*_*i*_^0^) normalized by the total MU (*MU*_*T*_):

1 $$ esf= \sin \left(\frac{M{U}_i^0}{M{U}_T}\times 2\pi \right),\kern0.37em i = 1,\  2,\  3,\overset{\dddot{}}{\ }N $$

where *N* is the total number of CPs. The *esf* simulates the potential error triggered by the gravitational effect during treatment. This form satisfies the criteria mentioned earlier, i.e., the maximum deviation occurs at gantry of 90° and 270° and no deviation at 0° and 180°, therefore such errors are undetectable by the conventional static QAs. The form is illustrated in Fig. 3.

Three types of errors are simulated: the gantry angle itself (a difference between expected and actual gantry angle during VMAT delivery), MLC position shift and output (MU) error as a function of gantry angle. The modification function of gantry angle, MLC position and MU of each CP are:

2 $$ {X}_{{}_i}^{\hbox{'}}={X}_i^0- Ma{g}_X\times esf $$

where *X* can be gantry angle *A*, or MLC position *P*, or linac output *MU*. Subscript *i* indicates the CP index. *X*_*i*_^0^ is the value in the original plan, and *X*_*i*_^'^ is the modified value. *Mag* is the error magnitude, ranging from 1° − 5° in gantry angle, 1–5 mm in leaf position and 1–5 % in linac output. Using equation (2), the gantry angle is modified to lag behind the planned angle from 0° to 180° and to exceed from 180° to 360°; the whole MLC bank is shifted towards the gravitational direction without changing the gap between; and the output error is negative from 0° to 180° while positive from 180° − 360°. While it is intuitive to attribute the sinusoidal form of error in gantry angle and MLC leaf position to the gravitational effect, the same form for linac output is purely speculative. A constant scaling error may be more likely; however, the analysis of the errors in such forms is rather straightforward and does not require the measurement to be performed on modified plans. We presented the analysis for both types of errors in the results.

In summary, the choice of sinusoidal function is due to the following considerations: 1. It could be interpreted as a function of gantry angle, which distinguishes VMAT form IMRT; 2. It has minimal magnitude at the usually checked positions; 3. It simulates the gravitational effect possible to trigger MLC and gantry errors. 4. It is of periodic form, and the accumulation over a whole period is zero; which is more difficult to be detected thus suitable for sensitivity analysis.

**Fig. 4:**
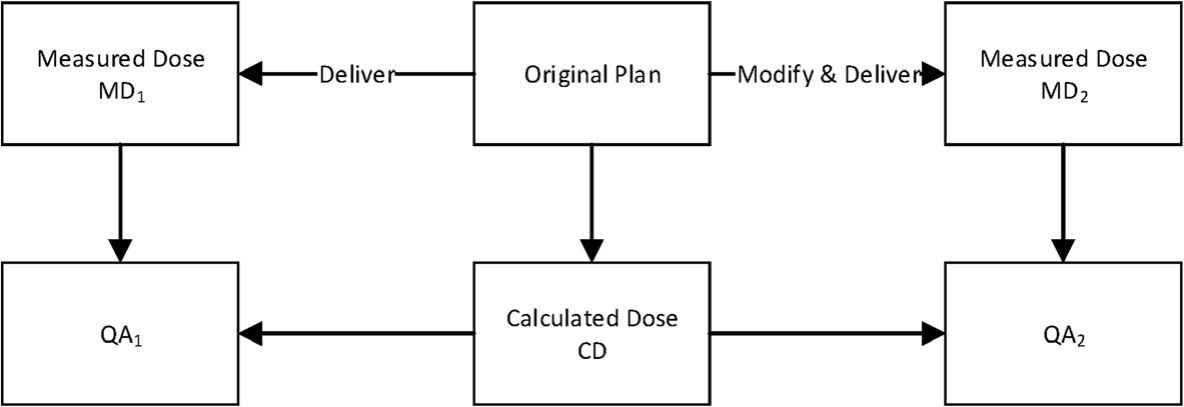
Flowchart of machine error simulation

The procedure of machine error simulation is shown in Fig. 4. Using the calculated dose (CD) and measured dose (MD_1_) of original plan, the QA procedure (QA_1_) is performed as reference. The machine error is simulated by performing the QA procedure (QA_2_) using the measured dose distribution (MD_2_) of the modified plan and originally calculated dose distribution (CD). Note that: for EPID technique, the CD used in QA_1_ and QA_2_ are calculated based on the delivery information of MD_1_ and MD_2_, respectively.

### Sensitivity analysis

Several approaches were taken to analyze the sensitivity qualitatively and quantitatively between the γ pass rates from QA_1_ and QA_2_, which include the overlap histogram, the gradient of average γ pass rates, and receiver operator characteristic (ROC) analysis.

#### Overlap of γ pass rate histograms

The overlap between the γ pass rates histograms of QA_1_ and QA_2_ can be used to evaluate sensitivity qualitatively. With the introduction of intended errors, γ pass rates are supposed to decrease, resulting in QA_1_ histogram centering at higher value and QA_2_ histogram at lower value. Intuitively, one can conclude that the less overlap between these two histograms, the more sensitive the system is. At one extreme, non-overlap indicates that modified plans can be totally separated from the original plans, thus the system is 100 % sensitive; another extreme is the total superposition, which suggests the modified plans are indistinguishable from original plans and the system is totally insensitive.

#### Gradient of γ pass rates vs error magnitude

For each type of error, the average *γ* pass rates from the cohort of plans are expected to decrease with the increasing error magnitude. A linear regression can be performed through least square fitting. The gradient or derivative, ∂, approximated by the slope of the linear function, is interpreted as the average decrease of *γ* pass rate per unit error. The steeper the line is, or the higher value the absolute gradient ∂ is, indicating the more sensitive the system is. One could use higher order (e.g., quadratic) function to fit the average *γ* pass rate vs the error magnitude, but it is found that the first order (linear) form is adequate for the purpose of comparison in this study.

#### ROC analysis

The receiver operator characteristic (ROC) analysis is a commonly used tool to quantitatively analyze the sensitivity [17]. The area under the ROC curve (AUC) can be used as an evaluation index. With an arbitrary threshold value set, the plans with γ pass rate greater than this threshold are categorized as “pass”, and the rest as “fail”. The ratio of number of “passed” original plans to the total original plans is defined as the true positive rate (TPR). Likewise, the ratio of number of “passed” modified plans to the total modified plans is the false positive rate (FPR). By continuously varying the threshold value, a set of corresponding TPR-FPR pairs are obtained, and they form the ROC curve. It is generally agreed that the higher value of AUC, the more sensitive the system is to the underlying variables.

#### Summary

Compared with other approaches, the advantage of ROC analysis is its independence from the arbitrarily chosen decision criteria (γ pass rate threshold, in this study), which is typically dependent on the institution. Therefore, the ROC analysis can be considered essentially free of the institutional bias. The sensitivity evaluation is simplified to the comparison of AUC values. For a commonly accepted AUC threshold value, one can derive the minimum detectable error magnitude. In this study, this AUC value was chosen to be 0.95. For histogram overlap analysis and gradient analysis, the DD, DTA and relative threshold dose value of all three systems are set to 2 %, 2 mm and 5 %. The ATA of EPID technique is set to 2°. For ROC analysis, the ATA is adjusted from 1° to 3° to test its impact on error sensitivity.

### Plan data

Fifteen full arcs from head & neck treatment plans were selected for the study. They were optimized for a Varian TrueBeam Linac with a Millennium 120 MLC on Eclipse treatment planning system (TPS, V11, Varian Medical System, Palo Alto, CA) using 6MV photon. The dose in the QA phantoms was calculated using the anisotropic analytical algorithm (AAA) with a grid size of 2.5 × 2.5 × 2.5 mm^3^. For ArcCheck the central plug was inserted during the measurement. Five magnitudes were simulated for each type of error, resulting in 15 modified plans in addition to the original plan. In total, the measurement and calculation of 240 plans were performed on each QA systems. The source to imager distance (SID) in EPID technique, and the source to axis distance (SAD) for both Delta^4^ and ArcCheck are 100 cm. All measurements for each arc were performed with the same setup, and the pass rates of original plans were all greater than 90 %, indicating that the setup errors were negligible. Furthermore, since the original and modified plans were delivered in the same setup condition, these minor setup errors were cancelled out when comparing the pass rates of QA_2_ to QA_1_, and should not affect the results of sensitivity analysis.

## Results

In the following, we first present the sample results of sensitivity with different analysis techniques, then the results for each type of machine errors in detail.

### Sample sensitivity analysis

**Fig. 5:**
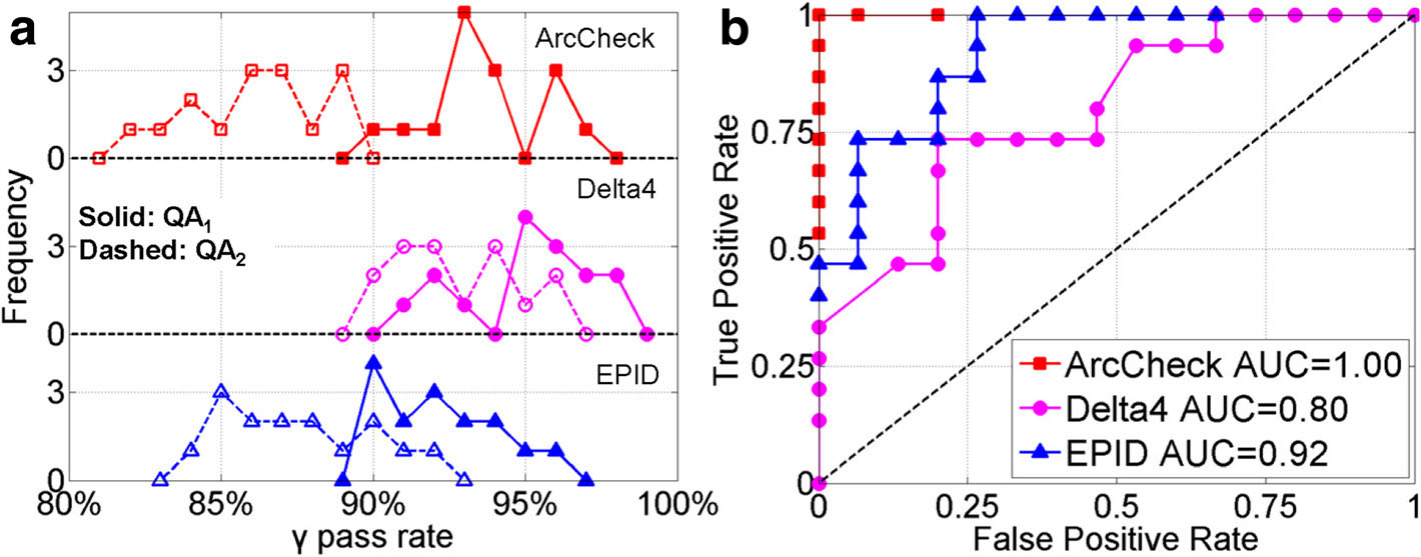
Sample results for 2° error in gantry angle. **a** pass rate histograms, **b** ROC analysis. The ATA of EPID is set to 2°

The original plans and the modified plans with 2° gantry error are used to demonstrate the analysis techniques. Figure 5(a) shows the γ pass rate histograms of QA_1_ and QA_2_. Comparing the overlap regions, we can see that ArcCheck has the least overlap, so we can assert qualitatively that ArcCheck system is more sensitive than the EPID method, which in turn is better than Delta^4^. Figure 5(b) demonstrates the ROC analysis with the AUC values also listed. The markers indicate the TPR-FPR pairs. The top left point (0, 1) represents the ideal case in which all original plans pass and all modified plans fail. The bottom left point (0, 0) and top right point (1, 1) represent “conservative” and “radical” decision making: by applying a too high or too low threshold, no plan or all plans pass. The diagonal line represents random guessing. Intuitively, the system is sensitive if its ROC curve is above the diagonal line. The sensitivity could also be compared quantitative: higher AUC value suggests higher sensitivity. From this, we can draw the same conclusion that ArcCheck is most sensitive and Delta^4^ is least sensitive.

##  Gantry angle sensitivity

**Fig. 6:**
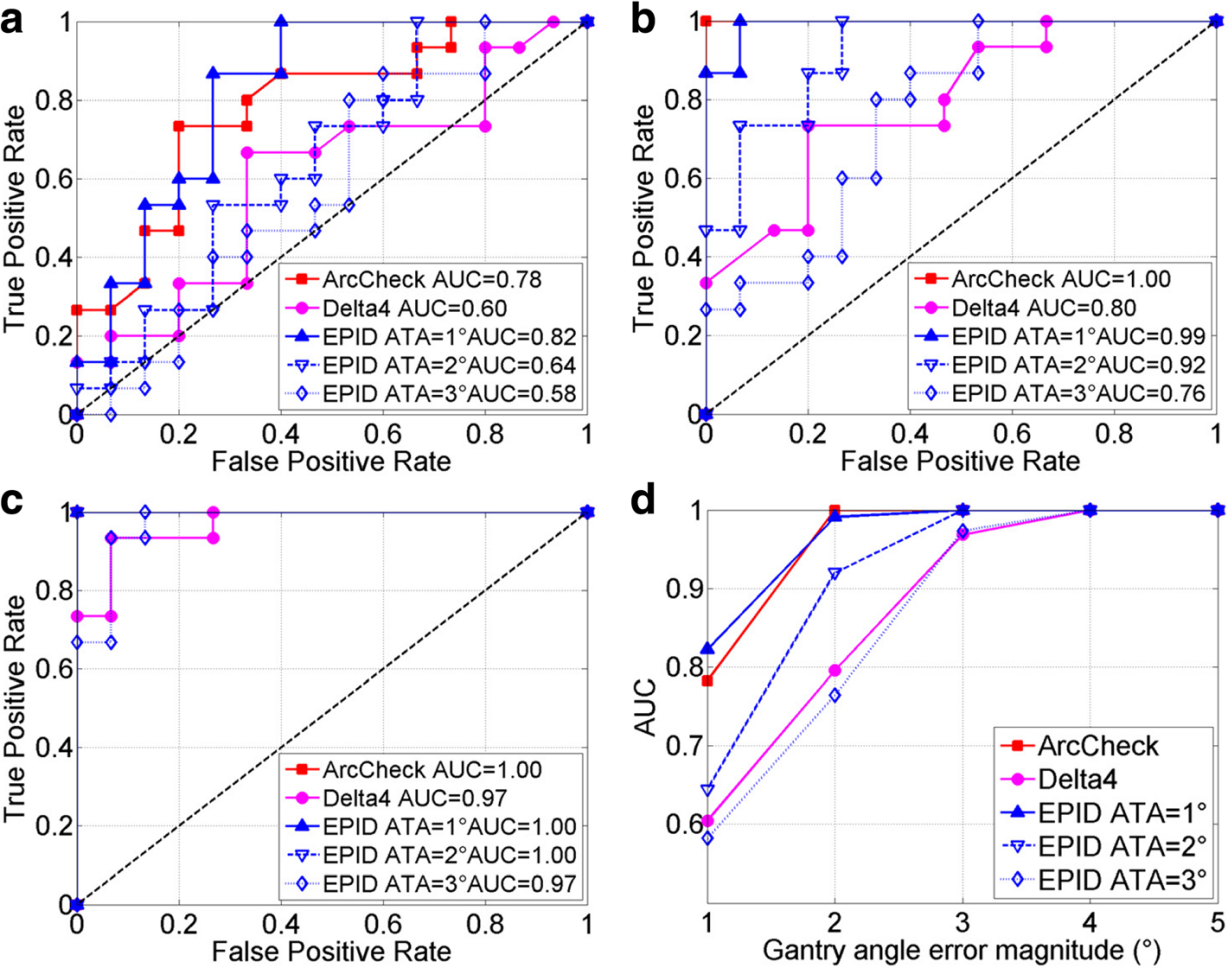
ROC analysis of gantry angle errors. **a**, **b** and **c** are for gantry angle error magnitude of 1°, 2° and 3°, respectively. **d** AUC vs. error magnitude. Curves with AUC of 1.0 reduce to a single point on upper left corner. 1°–3° ATA thresholds were chosen for EPID technique

**Fig. 7:**
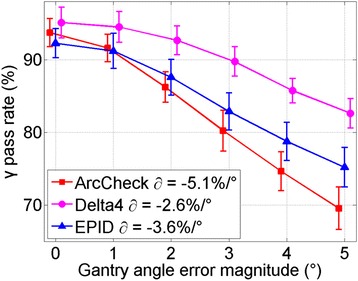
Pass rate gradient of error magnitude

Figure 6 shows the ROC analyses of the three systems: (a) to (c) are the ROC curves for 1° to 3° gantry error, (d) displays the AUC values as a function of error magnitude. The AUC values of ArcCheck, Delta^4^ and EPID are greater than 0.95 when gantry angle error exceeds 2°, 3° and 3° respectively. In general, ArcCheck outperforms EPID which in turn is more sensitive than Delta^4^. By setting ATA differently, the EPID sensitivity can be tuned to match ArcCheck or Delta^4^ system. Figure 7 shows the average γ pass rate as a function of error magnitude. The gradient ∂ from linear fit is also shown, supporting same conclusions.

### MLC position sensitivity

**Fig. 8:**
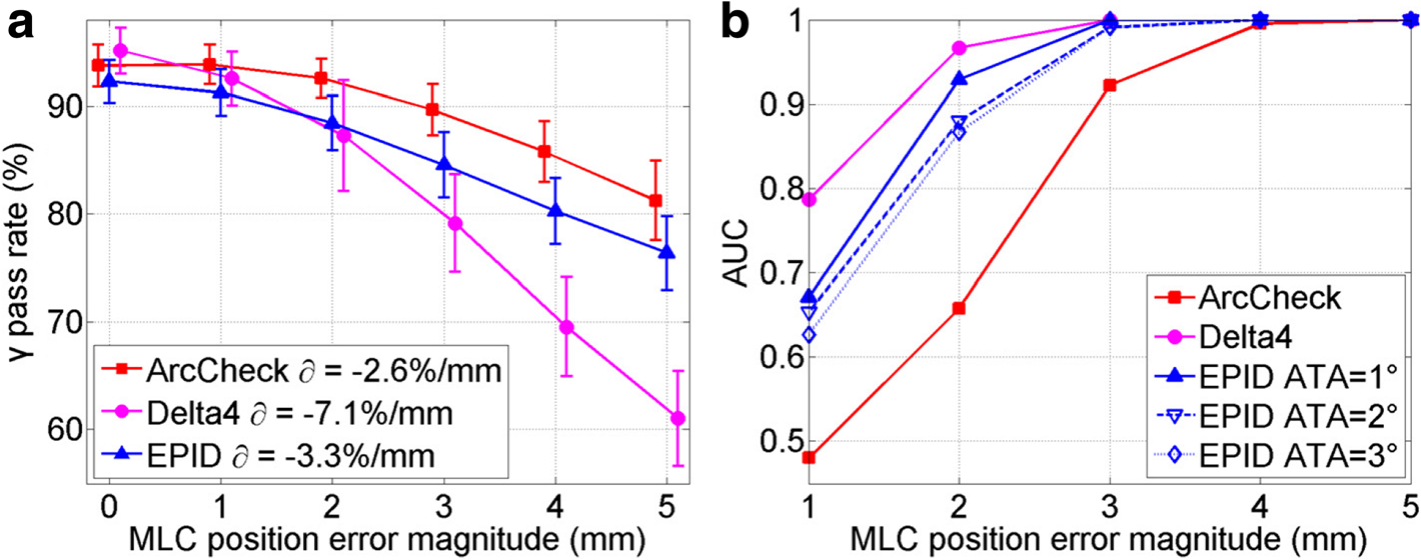
MLC leaf position error sensitivity analysis. **a** Gradient technique, **b** ROC analysis

Figure 8 shows results of sensitivity to MLC leaf position error. Figure 8(a) compares the average γ pass rate as a function of error magnitude. Using the AUC threshold of 0.95, the minimum detectable MLC error is 4, 2 and 3 mm for ArcCheck, Delta^4^ and EPID. Unlike the gantry angle error, now Delta^4^ is the most sensitive system with the largest absolute ∂, ArcCheck is the least, and EPID is in the middle. This is supported by the AUC curves plots in Fig. 8(b). Similarly, three ATA settings were used in EPID technique. However, they were not as effective as previous section, probably because that ATA could only be directly specified to gantry angle axis.

### Output error

**Fig. 9:**
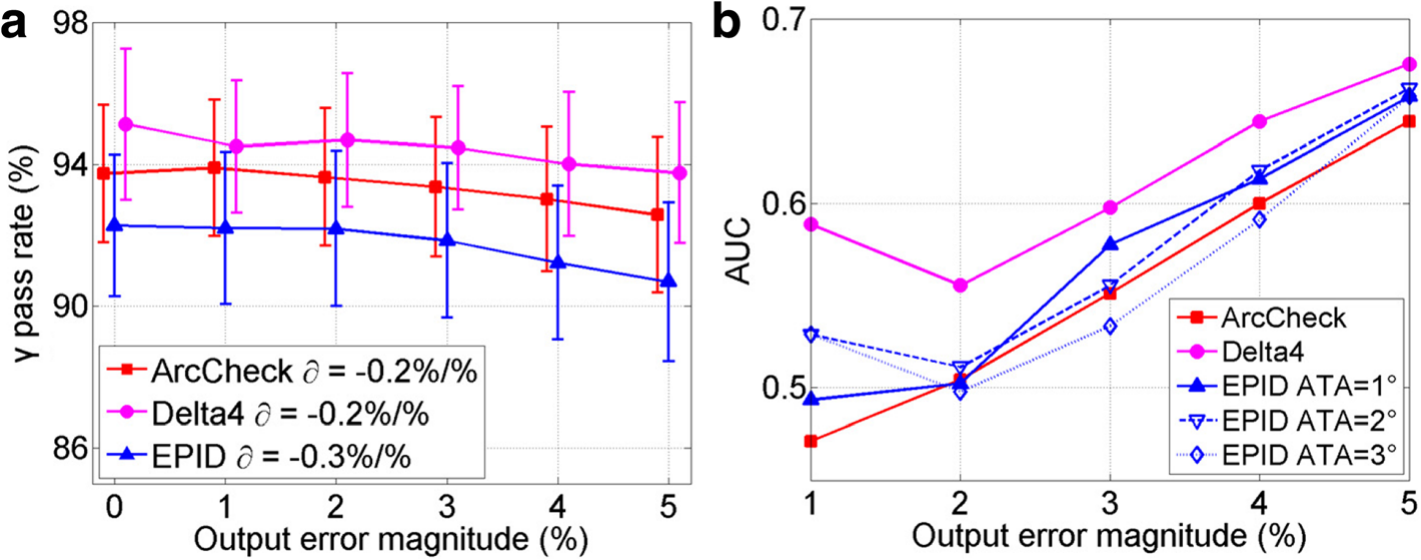
Linac output error sensitivity analysis. **a** Gradient technique, **b** AUC comparison for ROC analysis, notice reduced scale in vertical axis

The result for linac output error is shown in Fig. 9. While there is still the decrease of γ pass rate in Fig. 9(a), the slope is not as steep as in the cases for gantry angle and MLC position. Similarly, the increase of the AUC values in Fig. 9(b) is also much shallower. Therefore, it is not clear which system outperforms the others in detecting this type of errors, even though all shows some sensitivity.

The insensitivity may be caused by two factors. First, the *esf* in Eq. (1) is for a whole sinusoidal period, with positive values at one half period and negative in the other half. They may cancel out each other in the analysis of the full arc. Second, the error magnitude may be too small to be detectable.

**Fig. 10:**
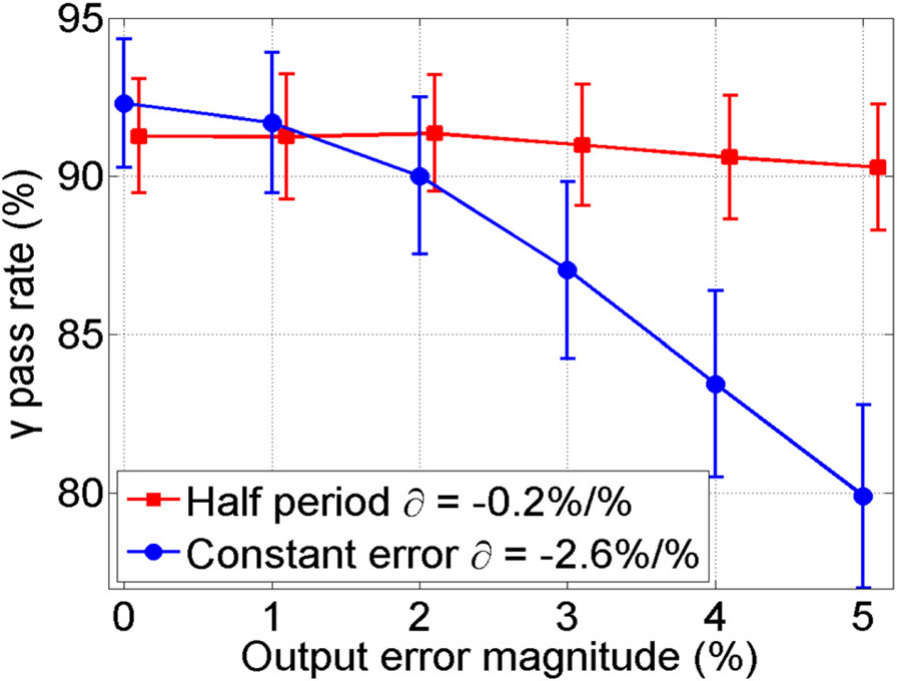
Sensitivity to output error for half arc and constant magnitude using gradient technique

To verify these two hypotheses, two experiments were further performed with EPID techniques. In the first experiment, γ analysis was performed only for the positive half arc. In the second one, a constant error (1–5 %) was applied for all CPs in the arc. The average γ pass rates are plotted in Fig. 10. The gradient ∂ is −0.2 %/% for half period simulation and −2.6 % for constant error. This confirmed that the insensitivity to output error in our sinusoidal form was not caused by the cancellation between positive and negative errors. Instead, the magnitude of simulated output error was too small to be detectable.

### ROC analysis using different criteria

The sensitivity analyses were also performed using different γ analysis criteria (2 %/3 mm, 3 %/2 mm and 3 %/3 mm) on gantry angle and MLC position errors. For each type of error, 5 error magnitudes were combined, and the resulting AUC values were listed in Table 1. The same trend as previous analyses was observed.

**Table 1:** ROC analyses using different γ criteria

Error type	QA system	2 %/2 mm	2 %/3 mm	3 %/2 mm	3 %/3 mm
Angle error	ArcCheck	0.956	0.943	0.955	0.948
	Delta4	0.874	0.809	0.844	0.799
	EPID 1°	0.963	0.971	0.969	0.987
	EPID 2°	0.913	0.930	0.916	0.946
	EPID 3°	0.864	0.880	0.870	0.907
MLC error	ArcCheck	0.811	0.833	0.829	0.840
	Delta4	0.951	0.923	0.946	0.916
	EPID 1°	0.920	0.908	0.928	0.918
	EPID 2°	0.905	0.891	0.911	0.907
	EPID 3°	0.897	0.887	0.908	0.907

AUC values of each system under different criteria are listed

## Discussions

In this study the potential machine errors were simulated based on the unique VMAT characteristic. The main difference from IMRT is the added gantry rotation, which is the reason why we investigated the gantry-angle related machine errors caused by the gantry inertial during rotation. The introduced MLC positional errors could be interpreted as the discrepancy caused by the gravitational force which can be reasonably assumed to be sinusoidal form. Since the gantry angles and dose outputs are usually checked daily at static angles such as 0°or 180°, but not dynamically, possible errors related to gantry angle and output during VMAT delivery must be in some form of periodical functions, and sinusoidal is a reasonable choice. There may be other forms of periodical functions that can be used. We believe the conclusion should not change with the choice of the functions.

The results presented here agree with the previous publications. Take Delta^4^ for example, Hauri et al. [8] reported that using 3 %/3 mm criteria, the average γ decrease of 2° sinusoidal gantry angle error is 0.1 %. The average decrease in our experiments is 0.6 % under the same criteria. Heilemann et al. [9] reported the average γ pass rate decreased 3.7 and 7.9 % with 2 mm sinusoidal MLC shift for head-and-neck and prostate plans using the criteria of 2 %/2 mm. In our study, the decrease was 7.9 %. Combining the results from the two studies, we can also come to the same conclusion that Delta^4^ is more sensitive to MLC shift than gantry angle error. This example also demonstrates that although many publications can be referred on this subject, a comprehensive understanding could not be obtained from any single publication.

Besides error sensitivity, the impact of machine errors on dose distribution is also an important aspect of VMAT QA investigation. Betzel et al. [18] simulated the machine errors in both IMRT and VMAT plans and compared the 3D patient dose distributions after feeding back the modified plans to the treatment planning system. They found that the VMAT plans is less susceptible than IMRT plans on the gantry angle error than the MLC error, which was supported by their Delta^4^ analysis. We want to point out that their analyses were performed on the target volumes, which were mostly deep-seated and close to isocenter. Their analysis was also similar to how Delta^4^ system works, i.e., on the 3D dose matrices (even though most of the Delta^4^ dose values were interpolated rather than directly measured). Therefore, the method was naturally less sensitive to the small gantry angle rotation errors. On the other hand, the ArcCheck has the detectors at a radius of 10.5 cm from the isocenter and its analysis is 2D in nature for the detector plane unfolded, which is sensitive to gantry angle rotations. In fact, its sensitivity should be proportional to the radial distance of the detectors to the isocenter. Therefore, their results are consistent with our findings, and further support the rationale of our study. It also shows that the two aspects of QA investigation are complementary and one study cannot replace the other. For this study, the focus is sensitivity comparison, and we plan to investigate the impact on dose distribution next.

Although γ analysis is widely used in clinical practice, recent publications have raised concerns on whether it is capable of catching planning or machine errors [14, 19, 20]. It was demonstrated that even with intentional errors, the pass rate may still be higher than 90 % [14, 19]. In this study, we simulated machine errors with varying magnitudes, and the sensitivity was investigated with γ gradient and ROC analysis. The sensitivity comparison was simplified to compare a single index. And more importantly, without the necessity of determining γ pass rate threshold in ROC technique, the analysis is free of subjective bias.

In addition, the γ analysis criteria (DTA, DD, ATA and dose threshold) directly affect the pass rate, these parameters may not have the same meaning in the three systems studied. For example, the DTA for ArcCheck stipulates the search range on the unfolded 2D plane. For Delta^4^, it is the distance of the interpolated 3D dosimetry matrix, while for EPID, it is the distance within each 2D PD plane. In this study, the goal is to evaluate each system’s sensitivity, the pass rates were compared with and without intentional errors (QA_1_ and QA_2_). Therefore, the effects of analysis criteria, as well as other factors like CP spacing, were cancelled out. Therefore, using the commonly used criteria (DTA: 2–3 mm, DD: 2–3 %, ATA: 1–3°, and dose threshold 5 %), the same sensitivity trend was observed.

We attribute the different sensitivity for these three systems to their different detector layout and the QA analysis performed. As shown in Fig. 1, the diode detectors of ArcCheck are embedded on a cylindrical surface, and only the dose on that 2D plane is measured and used in the γ analysis. While the detectors of Delta^4^ system are embedded on two near-orthogonal planes, a 3D dose matrix is generated for all points inside the phantom through the interpolation and used in γ analysis. For the EPID technique, the PD is measured with fine resolution in gantry angles. By stacking up the PD, a 3D dose matrix is obtained with the 3rd axis representing the gantry angle. Therefore, ArcCheck, Delta^4^ and EPID technique perform QA analysis on 2D, 3D and quasi-3D matrices, respectively.

These differences directly affect their performances in detecting machine errors. For example, gantry angle error leads to greater variations to the points on the cylinder surface than those in the inner region, while the MLC position error affects all points along the beam projection no matter on the surface or close to the isocenter. This explains that the ArcCheck is more sensitive to the gantry angle error and less sensitive to MLC position error because all the detectors are on the outer surface. If a hypothetical new version of ArcCheck is built with the cylinder diameter doubled, its sensitivity to gantry angle should also be doubled. For Delta^4^, only those points close to the outer surface will be affected by the gantry angle error greatly, but a large percentage of the points will be affected by MLC position error. Therefore Delta^4^ is the least sensitive to gantry angle error but most sensitive to MLC position error. If only the points in the outer shell of the phantom are used in the γ analysis, then its sensitivity can be expected to be similar to ArcCheck.

While errors of 5° or 5 mm are rare, they were included here to investigate the sensitivity. Such an error would be caught by the linac control software through treatment interlock under normal machine tolerances, but can potentially occur. We have shown that these QA systems are capable to detect errors of smaller magnitude. The actual machine errors occurring are the subject of other QA tasks. Another equally important issue is how these machine errors impact the treatment plans, i.e., the clinical relevance of these potential errors. The future work will include feeding back these modified plans to the planning system and evaluating the clinical consequences, which are likely treatment site specific.

One feature of EPID technique is the criteria on gantry angle can be explicitly specified in the analysis. Furthermore, EPID has the highest detector resolution and largest detector area so that field size up to 40 × 40 cm^2^ can be included in the QA. In comparison, the maximum field sizes detectable by ArcCheck and Delta^4^ are 21 × 21 cm^2^ and 22 × 20 cm^2^ in the central region.

## Conclusion

In summary, we compared three VMAT QA systems in terms of sensitivity to machine errors in gantry angle, MLC position and linac output. Based on the 0.95 criteria on the AUC value, the minimum reliably detectable gantry angle error of ArcCheck, Delta^4^ and EPID are 2°, 3° and 3°, respectively; and the minimum detectable MLC leaf position errors are 4 mm, 2 mm and 3 mm, respectively. ArcCheck is more sensitive to gantry angle and Delta^4^ is more sensitive to MLC position. The EPID technique’s sensitivity can match both systems by adjusting the extra angle-to-agreement parameters. In addition, EPID can handle the largest field size with highest spatial resolution and requires no extra phantom.

## Abbreviations

AAA: Anisotropic analytical algorithm
ATA: Angle to agreement
AUC: Area under the ROC curve
CD: Calculated dose
CP: Control point
DD: Dose difference
DTA: Distance-to-agreement
EPID: Electronic portal image device
*esf*: error simulation function
FPR: False positive rate
IMRT: Intensity modulated radiation therapy
MD: Measured dose
MU: Monitor unit
PD: Portal dose
QA: Quality assurance
ROC: Receiver operating characteristic
SAD: Source to axis distance
SID: Source to imager distance
TPR: True positive rate
TPS: Treatment planning system
VMAT: Volumetric modulated arc therapy
